# Role of T cells in a gp91^phox^ knockout murine model of acute allergic asthma

**DOI:** 10.1186/1710-1492-9-6

**Published:** 2013-02-07

**Authors:** Ena Ray Banerjee, William R Henderson

**Affiliations:** 1 Department of Medicine, Division of Allergy and Infectious Diseases, Center for Allergy and Inflammation, University of Washington, Room 254, 850 Republican Street, Seattle, WA 98109, USA; 2 Department of Zoology, University of Calcutta, 35 Ballygunge Circular Road, Kolkata 700019, West Bengal, India

## Abstract

**Objective:**

Molecular regulation of inflammation, especially, the role of effector cells in NADPH oxidase-mediated redox reactions for producing O_2_^-^ (superoxide anion) is a critical step. This study explores the roles of macrophages and neutrophils and their cross-talk with extra-cellular matrix components in the light of the role essayed by T cells. Materials and Methods and Treatment: To clarify the role of NADPH oxidase in the pathophysiology of T cell-initiatedmacrophage-associated allergic asthma, we induced allergen dependent inflammation in a gp91^*phox*^−/− SKO (single knockout) and a gp91^*phox*^−/− MMP-12−/− DKO (double knockout) mouse and analysed trafficking and functionality of various cell types, the T cell function and T cell-macrophage interaction being given special emphasis.

**Results:**

Composite asthma symptoms expressed in a more aggravated manner in both the KO (SKO and DKO) mice compared to WT indicating that some redundancy may exist in the response pathways of gp91phox and MMP-12. On the one hand, upregulation in macrophage functions such as proliferation, mixed lymphocyte reaction, and MCP-1 directed chemotaxis, may indicate that a regulatory cross-talk is switched on between T cell and macrophage and on the other, downregulation of respiratory burst response hints at a dichotomy in their signaling pathways. Increased B7.1 but reduced B7.2 and MHC class II expression on KO alveolar macrophages may suggest that a switching on-off mechanism is operative where alteration of co-stimulatory molecule expression selectively activating T cell is a critical step.

**Inference:**

T cell mediated functions such as Th2 cytokine secretion, and T cell proliferation in response to OVA were upregulated synchronous with the overall robustness of the asthma phenotype.

**Conclusions:**

As far as cell-cell interaction is concerned, the data is indicative of the existence of a plethora of networks where molecular switches may exist that selectively induce activation and deactivation of regulatory pathways that ultimately manifest in the overall response. gp91^*phox*^ and MMP-12 either redundantly or synergistically but not additively, provide a regulatory checkpoint for restricting T cell cross-talk with macrophages and keep excessive tissue damage and ECM degradation during acute allergic inflammation under control.

## Introduction

The production of superoxide anions (O_2_^-^) by neutrophils and other phagocytes is an important step in our body's innate immune response. [1,2]. These act as microbicidal agents and kill invading micro-organisms either directly or through the activation of proteases [3,4]. O_2_^-^ is produced by the NADPH oxidase, a multi-protein enzyme complex, which is inactive in resting phagocytes, but becomes activated after interaction of the phagocyte with pathogens and their subsequent engulfment in the phagosome [5]. Defects in the function of the NADPH oxidase result in a severe immunodeficiency, and individuals suffering from CGD (chronic granulomatous disease), a rare genetic disorder that is caused by mutations in NADPH oxidase genes, are highly susceptible to frequent and often life-threatening infections by bacteria and fungi [6,7].

NADPH oxidase, the primary source of reactive oxygen species is a strong candidate for the development of therapeutic agents to ameliorate inflammation and end-organ damage [8,9]. Additionally, the study of cytochrome isolated from patients with X-linked CGD has contributed to our current understanding of its function [10,11].

Involvement of the gp91phox subunit in oxidative burst response by PMNs as well as Macrophages is not clear. Macrophages use a membrane-associated NADPH oxidase to generate an array of oxidizing intermediates. In some studies, it has been demonstrated that oxidants potently and efficiently inactivate matrilysin (MMP-7) by cross-linking adjacent tryptophan-glycine residues within the catalytic domain of the enzyme. These *in vitro* observations suggest that MMP inactivation can occur on or near phagocytes that produce both MMPs and reactive intermediates. In the absence of reactive intermediates, unrestrained proteolytic activity might lead to detrimental tissue damage. Indeed, inherited deficiency of gp91^*phox*^, a phagocyte-specific component of the NADPH oxidase required for oxidant production, and targeted deletion of its mouse homologue result in granuloma formation and excessive tissue destruction [12].

This study addresses for the first time the relationship between gp91phox and MMP-12 in the development of T cell mediated acute allergic asthma in a mouse model using genetic knockout mice, gp91phox−/− which will be referred to as NOX−/− or SKO and MMP-12-NOX DKO. The study focuses on the cross-talk between T cells and macrophages and shows that gp91phox most likely has a regulatory role in the onset and maintenance of the composite asthma phenotype in mouse and deletion of gp91phox may alter expression of co-stimulatory/co-inhibitory molecules B7.1 (increased) and B7.2 (decreased) and MHCII expression (increased) which may explain the mechanism by which macrophages despite increased migration to the inflammatory foci *in vivo* and increased migration in a chemotaxis chamber to MCP-1, and enhanced proliferation to syngeneic or allogeneic stimulus *in vitro*, fail to execute oxidative burst response. MMP-12 seems to be either redundant, not contributing to the overall asthma phenotype or has a synergistic (not additive) role in the process.

## Materials and Methods

### Mice

Both gp91^phox−/−^ mice [13,14] were on a C57Bl/6J background and had been outcrossed and then intercrossed for three generations to generate animals deficient in both genes. C57BL6 mice (Taconic) were used as the control group and are called wildtype. In total the following number of animals were used in each group: WT (14), NOX−/− (14), MMP-12NOX−/−(16) in the control group and WT (16), NOX−/− (15), MMP-12NOX−/−(14).

### Allergen sensitization and challenge

Mice were sensitized and later challenged with OVA (Thermo Scientific Pierce Protein Research Products, Rockford, IL) as described previously [15].

### Tissue analyses

The mouse underwent exsanguination by intra-orbital arterial bleeding and cells, obtained by bronchoalveolar lavage and those from lung parenchyma (obtained by lung mincing and digestion was performed after lavage as described previously [15] with 100u/ml collagenase for 1 hr at 37°C, and filtered through a 60# sieve (-Aldrich Corporation, St. Louis, Sigma) were evaluated after air drying, by staining with Wright-Giemsa (Biochemical Sciences Inc, Swedesboro, NJ) and their differential count was taken under a light microscope at 40X magnification. Cell number refers to that obtained from lavage of both lungs/mouse. In addition, cells from hemolysed peripheral blood (PB), bone marrow( BM), bronchoalveolar lavage (BAL), lung parenchyma (LP), spleen, mesenteric lymph nodes (MLN), cervical lymph nodes (CLN), axillary lymph nodes (LNX) and inguinal lymph nodes (LNI) were analyzed on a FACSCalibur (BD Immunocytometry Systems, San Jose, CA) by using the CELLQuest program. Staining was performed by using antibodies conjugated to fluorescin isothiocyanate (FITC), phycoerythrin (PE), allophucocyanin (APC), Peridinin Chlorophyll Protein (Per CP-Cy5.5) and Cy-chrome ( PE-Cy5 and PE-Cy7). The following BD pharmingen (San Diego, CA) antibodies were used for cell surface staining : APC-conjugated CD45 (30F-11), FITC-conjugated CD3(145-2C11), PE-Cy5 conjugated CD4 (RM4-5),PE-conjugated CD45RC (DNL-1.9), APC-conjugated CD8(53–6.7), PE-Cy5 conjugated B220 (RA3-6B2), FITC-conjugated IgM, PE-conjugated CD19 (ID3), PE-conjugated CD21(7G6), FITC-conjugated CD23 (B3B4), APC-conjugated GR-1(RB6-8C5), and PE-conjugated Mac1(M1/70). PE-Cy5 conjugated F4/80 (Cl:A3-1(F4/80)) was obtained from Serotec Ltd., Oxford, UK. PE-conjugated anti-α4 integrin (PS2) and anti-VCAM-1(M/K-2) was from Southern Biotechnology, Birmingham, Ala. Irrelevant isotype-matched antibodies were used as controls.

### Chemotaxis assay

Chemotaxis assay was performed with 10 million macrophages pooled from 4 mice/experimental group. Macrophages were prepared by adhering BALf cells in high glucose medium for 2 hours followed by detachment by mechanical scraping and resuspension in Phenol red-free high glucose DMEM (Gibco) with 5% FBS with 0.5μg/ml Calcein-AM (1:2000 dilution) and incubation for 20 min at 37°C. MCP-1 at dilutions ranging from 0.1-25mM were used and 15 mM was taken to be the optimum dose. 96 well Neuroprobe CTX plates (Chemicon, Temecula,CA) were used. 29μl MCP-1 (15mM) was added as a single convex drop and the polycarbonate filter placed gently over it and incubated at 37°C for 30 min. Cell suspension was added in designated slots over the filter membrane also in 29μl volume. The chamber was incubated at 37°C in humidified CO_2_ incubator for 2h. Excess cells were wiped off with kimwipes at the end of the incubation period. Migrated cells were quantified by fluorescence (excitation at 488 nm, emission at 520 nm) using a Victor 3V (Perkin Elmer laboratories) using a Wallac1420 software.

### Oxidative burst reposnse

Alveolar leukocytes (0.5 × 10^6^ cells) were stained with F4/80-Cy-Chrome and Gr1-APC for 30 min on ice, washed in PBS, warmed up at 37°C for 5 min and loaded with 5mM dihydrorhodamine 123 (Molecular Probes, Eugene, OR). After 10 min at 370C, cells were split in two equal aliquots, and PMA (Sigma, St. Louis, MO) was added to one aliquot at final concentration of 1mM. After 10 min incubation cells were washed in ice-cold PBS and immediately subjected to FACS analysis. Cells were gated on neutrophils (Gr1hi), or monocyte/macrophages (F4/80+) and percentage of cells positive for dihydrorhodamine 123 fluorescence with or without PMA treatment was determined for each gate.

### Real time-PCR analysis

For real time PCR analysis of mRNA expression of particular genes in differentiating human ESC as well as in the lungs of recipient animals in transplantation experiments, total RNA was extracted from cells (<500/sample) by PicoPure RNA isolation kit from Arcturus, Mountainview, CA) and those from lungs (kept in RNA later (Ambion) at -80°C), by RNA extraction kit (RNeasy) from Qiagen and cDNA made from it using superscript III system from Invitrogen (Life Technologies, Grand Island, NY). The PCR reaction solution contained 0.5 μg of total RNA, 6-mM magnesium chloride, and 0.5-μM of each primer (primer oligo sequences are in Table 1). Other components in the reverse transcriptase–PCR master mix included buffer, enzyme, SYBR Green I, and deoxyribonucleotide triphosphate. For reverse transcription, the 20 μL of reaction capillaries were incubated at 50°C for 2 min followed by a denaturation at 95°C for 10 min. Polymerase chain reaction by an initial denaturation at 95°C for 15 s and then annealing at 60°C 1 min, repeat 45 cycles. Finally, a melting curve analysis was perfomed by following the final cycle with incubation at 95°C for 15 s, at 60°C for 15 s, then 95°C for 15 s. Negative control samples for the reverse transcriptase–PCR analysis, which contained all reaction components except RNA, were performed simultaneously to determine when the nonspecific exponential amplification cycle number was reached. Forward and reverse primers are as in Table 1 and were synthesized by the University of Washington Biochemistry services using the Primer Express software. PCR was carried out using the comparative Ct method (Applied Biosystems software) with SYBR Green PCRcore reagents (Applied Biosystems Life Technologies) and anlysed using Applied Biosystems 7900HT Real-Time PCR System software SDS 2.2.1.

**Table 1:** List of mouse primers for real time PCR

**Cell marker**	**Gene**	**Forward primer**	**Reverse primer**
House- keeping genes	GAPDH	CGTCCCGTAGACAAAATGGT	TCAATGAAGGGGTCGTTGAT
	β-actin	GTGGGCCGCTCTAGGCACCAA	CTCTTTGATGTCACGCACGATTTC
Cytokine genes	IFN-γ	GCGTCATTGAATCACACCTG	TGAGCTCATTGAATGCTTGG
	IL-1α	TCAAGATGGCCAAAGTTCCT	TGCAAGTCTCATGAAGTGAGC
	IL-1β	TGAAGCAGCTATGGCAACTG	GGGTCCGTCAACTTCAAAGA
	IL-2	AACCTGAAACTCCCCAGGAT	CGCAGAGGTCCAAGTTCATC
	IL-3	CCGTTTAACCAGAACGTTGAA	CCACGAATTTGGACAGGTTT
	IL-4	GGCATTTTGAACGAGGTCAC	AAATATGCGAAGCACCTTGG
	IL-5	ATGGAGATTCCCATGAGCAC	AGCCCCTGAAAGATTTCTCC
	IL-6	AACGATGATGCACTTGCAGA	GGTACTCCAGAAGACCAGAGGA
	IL-10	TGAATTCCCTGGGTGAGAAG	TGGCCTTGTAGACACCTTGG
	IL-12β	ATCGTTTTGCTGGTGTCTCC	CATCTTCTTCAGGCGTGTCA
	IL-13	CCTCTGACCCTTAAGGAGCTT	ATGTTGGTCAGGGAATCCAG
	MCP-3	TCTGTGCCTGCTGCTCATAG	CTTTGGAGTTGGGGTTTTCA
Growth factor genes	TGFβ2	GGAGGTTTATAAAATCGACATGC	GGCATATGTAGAGGTGCCATC
	VEGFa	TACCTCCACCATGCCAAG	TGGTAGACATCCATGAACTTGA
	VEGFb	GGCTTAGAGCTCAACCCAGA	TGGAAAGCAGCTTGTCACTTT
	VEGFc	GGGAAGAAGTTCCACCATCA	TCGCACACGGTCTTCTGTAA

### Statistical analysis

Statistical differences among samples were tested by Student *t* test. *P* value less than 0.05 was considered statistically significant.

## Results

### Inflammatory recruitment index in KO mice post-OVA compared to WT

The various nuances of allergic asthma developed fully and more exaggeratedly in both gp91^phox^−/− and MMP-12-gp91^phox^ double knockout mice post OVA treatment compared to WT as described in detail in [16]. Absolute inflammatory migration profile measured by increase in cell number in tissues versus their sites of poiesis and circulation, i.e.bone marrow and peripheral blood respectively was increased (Table 2). In bone marrow, NOX−/− post OVA has 1.4-fold more cells (data not shown), in peripheral blood, 1.3-fold more cells, spleen had 1.3-fold more cells (data not shown), lung parenchyma had 1.8-fold more cells, and BALf: 2-fold more cells compared to post-OVA WT. Of note, 2.4-fold more PMNs, 1.96-fold more B lymphocytes, 5-fold more eosinophils in post-OVA NOX−/− and DKO compared to post-OVA WT BAlf was found [17].

**Table 2:** Recruitment index

		**T cells**	**B cells**	**Macs**	**PMNs**	**Eos**	**Basophils**
Lungs	WO	0.6	0.015	0.22	0.18	3	0.04
	NOXO	0.6	0.19*	1.3*	0.49*	3.8	0.16*
	DKOO	0.58	0.05*	1.1*	0.44*	3.4	0.13*
BALf	WO	0.22	0.25	0.77	0.107	5.75	0.03
	NOXO	0.32*	0.17	0.85	0.27	6.3	0.09
	DKOO	0.51*	0.16	0.78	0.26	5.8	0.08

Upregulation of inflammatory recruitment index in lung and airways of KO post-OVA. Cell number was counted using a Z1 particle counter from Beckman Coulter. Blood (PB) was obtained by infra-orbital bleeding and extrapolated to a 2 ml volume as the total volume of blood in a 20gm mouse, from the volume of blood actually obtained, perfused lung was minced and digested with collagenase IV and single cell suspension made of both lungs, and brochoalveolar lavage fluid (BALf) was obtained from both lungs, and the cell numbers counted. Recruitment index was calculated as a ratio of number of cells in lung parenchyma tissue versus number of the same cell subtypes in peripheral circulating blood and similarly in lung interstitium versus circulating cells. This denotes the fraction of inflammatory cells recruited from blood and migrated to lung. The data shown have been derived from 3 independent experiments and expressed as mean values ± SEM. *denotes p value<0.01 compared to post-OVA wildtype values. Abbreviations used are: WT=wildtype, NOX=gp91phox−/−, DKO=gp91phox-MMP-12 double knockout, WA=WT+alum, WO=WT+OVA, NOXA=gp91phox−/−+alum, NOXO=gp91phox−/−+OVA, DKOA= gp91phox-MMP-12 double knockout+alum, DKOO= gp91phox-MMP-12 double knockout+OVA.

### Over-expression of Th2 cytokine gene expression in the lung

**Figure 1:**
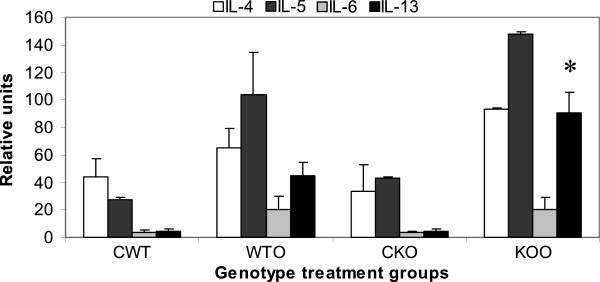
**Alteration of TH cytokine gene expression in lung.** Real time PCR analysis was used to quantitate expression of mRNA for the particular genes as calculated by relative index of Ct values normalized to GAPDH by real time PCR. PCR was carried out using the comparative Ct method (Applied Biosystems software) with SYBR Green PCRcore reagents (Applied Biosystems) and anlysed using Applied Biosystems 7900HT Real-Time PCR System software SDS 2.2.1. All primers used were specific to mouse. Data expressed here are mean ± SEM. n = 5/group. While all other Th2 cytokine levels were comparable to WT+OVA, IL-13 concentration was increased 2.7-fold over post-OVA WT values. Abbreviations used are: CWT = saline treated control wildtype, WTO = WT+OVA, CKO = gp91phox−/−+alum, KOO = gp91phox−/−+OVA. MMP-12.

Cytokine concentrations present in BALf was measured by ELISA and protein concentrations were presented in [16]. Table 3 shows that actual mRNA upregulation was 1.4-fold for IL-4 gene and 1.9-fold for IL-13 genes in the lung parenchyma tissue which are Th2 specific. There was also upregulation in IL-1α, IL-10 and IL-12α, the dignificance of which is not clear at this point. Figure 1 shows 2.75-fold increase in IL-13 gene expression in gp91phox−/− mice post-OVA compared to WT post-OVA. IL-4 was increased All other Th2 cytokines showed values similar to post-OVA WT BALf. Overall, IL-4: NOX−/− post OVA has 1.2-fold more protein and 2-fold more mRNA; IL-5: NOX−/− post OVA has 2-fold more protein and 2.8-fold more mRNA; IL-13: NOX−/− post OVA has 3-fold more protein and 5.6-fold more mRNA. Therefore, both by protein concentration and mRNA expression, Th2 cytokines show manifold increase in gp91phox−/− post-OVA compared to WT.

**Table 3:** Enhanced cytokine gene expression in KO lung

	**IFN-γ**	**IL-1α**	**IL-1 β**	**IL-2**	**IL-4**	**IL-5**	**IL-6**	**IL-10**	**IL-12β**	**IL-13**
**CWT**	4.67±1.70	71.27±6.47	3.79±0.32	2.57±0.02	44.26±12.70	27.15±1.70	3.59±1.65	4.21±2.60	7.27±5.25	4.72±1.38
**WTO**	4.96±1.85	411.67±2.46	21.17±8.11	65.56±13.28	65.42±13.58	103.45±30.68	20.04±9.80	23.61±3.66	21421.95±15756.71	45.15±9.45
**CKO**	1.18±0.271	62.81±0.68	6.20±1.58	2.65±1.61	33.72±19.35	43.12±0.90	3.74±0.96	1.76±0.65	29.23±21.91	4.68±1.32
**KOO**	2.06±0.60	6037.51+5.50	33.65±1.74	8.63+1.72	92.78+1.02	147.74±1.42	20.58±8.28	134.96+3.43	306948.33+107868.44	90.59+14.51

Real time PCR analysis shows expression of mRNA for the particular genes as calculated by relative index of Ct values normalized to GAPDH by real time PCR. PCR was carried out using the comparative Ct method (Applied Biosystems software) with SYBR Green PCRcore reagents (Applied Biosystems) and anlysed using Applied Biosystems 7900HT Real-Time PCR System software SDS 2.2.1. All primers used were specific to mouse. The graphs in * denotes p value <0.01 compared to bleo-treated untransplanted group. Mean denotes the avergae of 2 independent experiments (n=4/group). Underlined numbers denote mRNA expression in relative units normalized to mouse GAPDH that have p value<0.05 compared to wildtype post-OVA. Abbreviations used are: cWT= saline treated wildtype, WTO=wildtype post-OVA treatment, CKO=saline treated gp91phox knockout, KOO= OVA treated gp91phox knockout, CKO=OVA treated control.

### Chemokine and growth factor gene expression

MCP-3 is a known macrophage chemotactic protein. Its gene expression was found to be upregulated manifolds both in the saline treated control lung as well as post-OVA. The reson for this may be that in the absence of gp91phox, there is spontaneous upregulation of the chemokine gene. VEGFb was upregulated by 2.2-fold. Surprisingly, there was downregulation of TGFβ. MMP-12 (Table 4).

**Table 4:** Alteration in growth factor genes in KO lung post-OVA

	**MCP-3**	**TGFβ2**	**VEGFa**	**VEGFb**	**VEGFc**
**CWT**	4.63±1.63	18.03±15.34	1973.06±1549.62	176.39±2.24z	2283.58±1671.63
**WTO**	7.14±1.94	44.67±22.84	4260.86±1982.14	334.59±3.56	4500.67±1827.83
**CKO**	20901.40±6361.97	32.88±9.71	34.40±13.79	106.73±80.67	15.11±0.97
KOO	166575.87±119300.10	28.30±23.65	297.97±234.29	744.17±61.60	35.15±11.41

Real time PCR data shows expression of mRNA for the particular genes as calculated by relative index of Ct values normalized to GAPDH by real time PCR. PCR was carried out using the comparative Ct method (Applied Biosystems software) with SYBR Green PCRcore reagents (Applied Biosystems) and anlysed using Applied Biosystems 7900HT Real-Time PCR System software SDS 2.2.1. All primers used were specific to mouse. The underlined numbers p value <0.01 compared to OVA -treated wildtype group. Abbreviations used are: cWT= saline treated wildtype, WTO=wildtype post-OVA treatment, CKO=saline treated gp91phox knockout, KOO= CKO=OVA treated gp91phox knockout.

### Expression of Rho kinase and MMPs associated with inflammation

**Figure 2:**
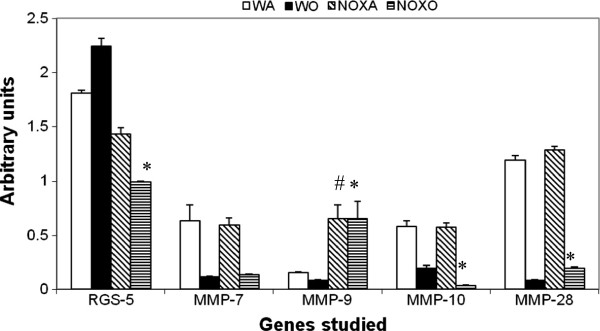
**Downregulation Rho kinase RGS-5 and MMP10 but upregulation of MMP9 and MMP28 genes in KO lung.** Real time PCR analysis was used to quantitate expression of mRNA for the particular genes as calculated by relative index of Ct values normalized to GAPDH by real time PCR. PCR was carried out using the comparative Ct method (Applied Biosystems software) with SYBR Green PCRcore reagents (Applied Biosystems) and anlysed using Applied Biosystems 7900HT Real-Time PCR System software SDS 2.2.1. All primers used were specific to mouse. * denotes p value<0.01 compared to WT+OVA values. # denotes p value<0.01 compared to WT+alum (control baseline values). n=5/group pooled from 2 experiments. Expression of the gene of interest was expressed in relative values normalized to the values obtained for mouse GAPDH. Compared to post-OVA WT values, Rho kinase RGS-5 mRNA was decreased 2.3-folds and MMp-10 3-fold, while MMP-9 was increased 8-fold and MMP-28 increased 2.3-fold (*denotes p value<0.05 compared to post-OVA WT).

**Figure 3:**
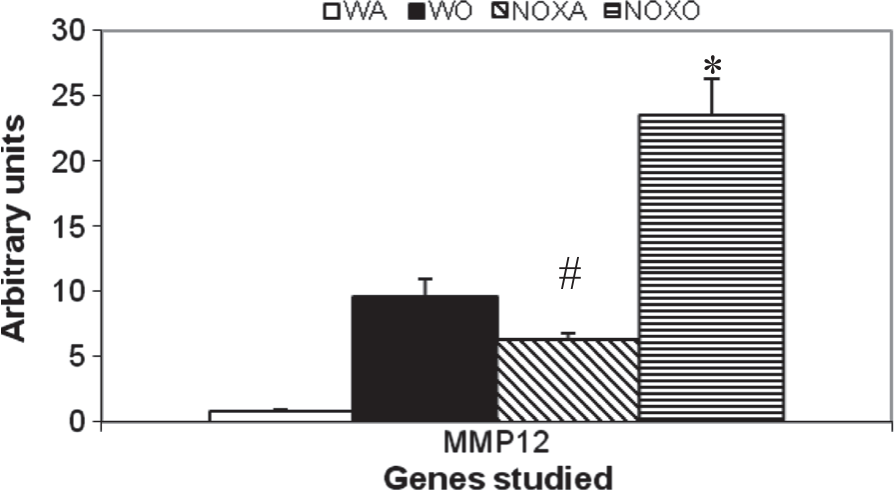
**Upregulation of MMP-12 gene in post-OVA in KO lung.** Real time PCR analysis was used to quantitate expression of mRNA for the particular genes as calculated by relative index of Ct values normalized to GAPDH by real time PCR. PCR was carried out using the comparative Ct method (Applied Biosystems software) with SYBR Green PCRcore reagents (Applied Biosystems) and anlysed using Applied Biosystems 7900HT Real-Time PCR System software SDS 2.2.1. All primers used were specific to mouse. * denotes p value<0.01 compared to WT+OVA values. # denotes p value<0.01 compared to WT+alum (control baseline values). n=5/group pooled from 2 experiments. Expression of the gene of interest was expressed in relative values normalized to the values obtained for mouse GAPDH. There was 10-fold increase in the gene expression of MMP-12 in OVA-treated WT vs. control WT whereas saline-treated NOX KO lungs showed 6.5-fold increase over untreated control mouse lung. However, post-OVA treatment, NOX KO mouse lung showed MMP-12 shooting up 4.7-fold over untreated KO. # denotes p value <0.01 compared to control untreated, * denotes p value <0.01 compares untreated KO versus OVA-treated KO lung.

Since this is an inflammatory response to the allergen, we hypothesized that other pro-inflammatory kinases and proteases may also be intimately involved in the pathway. Figure 2 and 3 shows RT-PCR analysis of gene expression of Rho kinase, known to have anti-inflammatory functions. Genes for MMP7, 10 and 28 were similarly downregulated as well.

### Functionality of T cells

**Figure 4:**
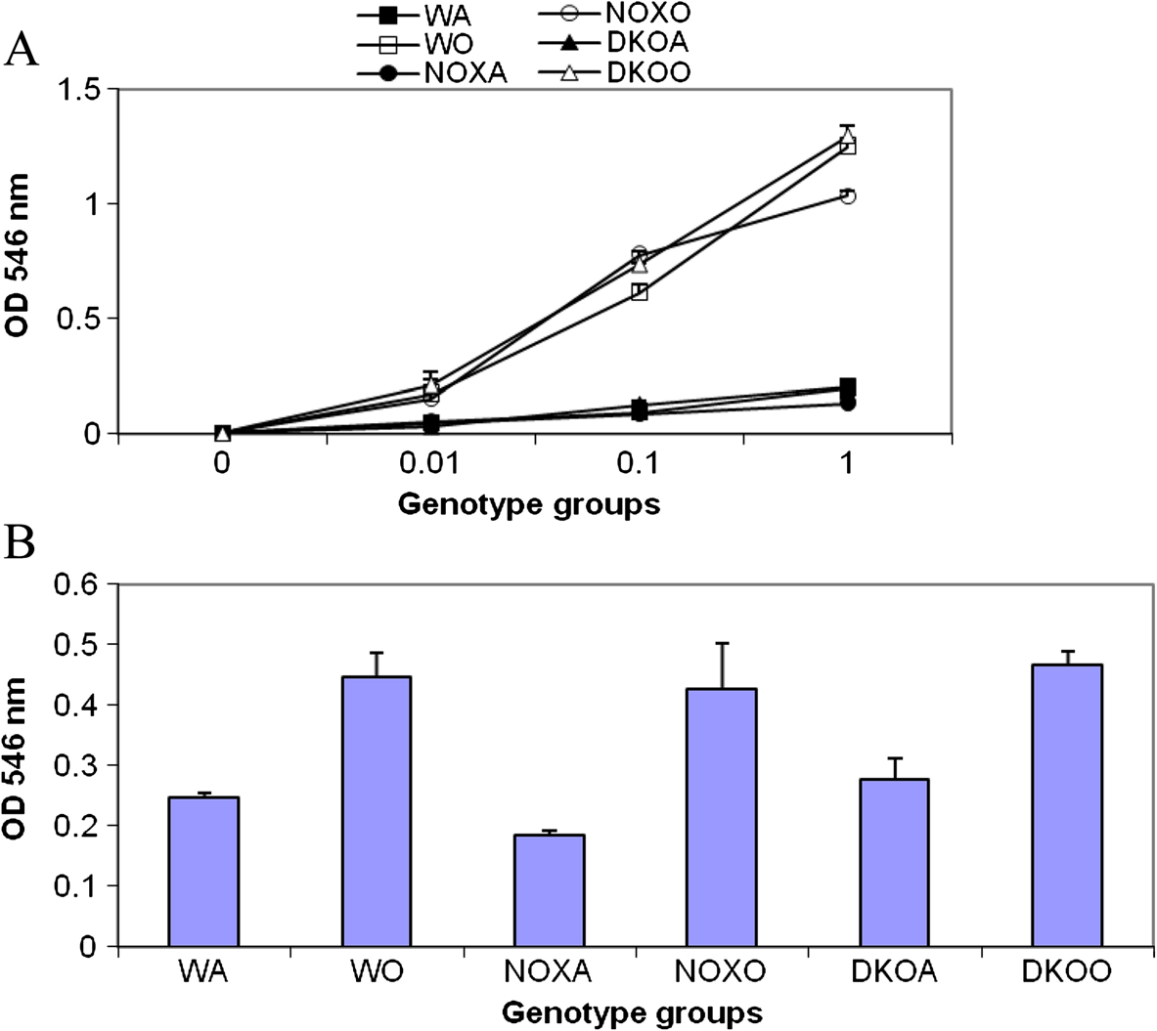
**T cells response in WT vs. KO mice. A.** Splenocytes from control (saline treated) and OVA treated mice were made into single cell suspensions in DMEM+10% heat-inactivated FCS. 0.1 million cells were plated per well without and with increasing concentrations of anti-CD3 antibody and a constant concentration of anti-CD28 antibody (1μg/ml) and cultured for 3 days. **B.** 1μM PMA and 10ng/ml ionomycin was used to stimulate splenocytes from the above experimental mice and proliferation measured after 3 days. To measure proliferation, MTT assay called CellTiter96 (Promega) was used. OD 546 nm is directly proportional to the number of cells in culture. Abbreviations used are: WT=wildtype, NOX=gp91phox−/−, DKO=gp91phox-MMP-12 double knockout, WA=WT+alum, WO=WT+OVA, NOXA=gp91phox−/−+alum, NOXO=gp91phox−/−+OVA, DKOA= gp91phox-MMP-12 double knockout+alum, DKOO= gp91phox-MMP-12 double knockout+OVA. Data presented are average of 3 independent experiments ± SEM. (n=5/group).

Proliferation of MACS-purified (>86-92%) CD4+ and CD8+ splenocytes by MTT incorporation assay and OD measurement at 545nm of anti CD3/CD28 (0.01-1ug/ml)induced proliferation of CD4+ shows a 8.4-folds increase in post-OVA WT compared to a 7.4-fold increase in both KO mice. In CD8+ while post-OVA WT increased by 6.4-fold, the KO mice showed 7.9-fold increase compared to the corresponding saline treated mice. With PMA/ionomycin (10ng/ml), CD4+ (post-OVA WT was 2.3-fold more than that in NOX−/−) while CD8+ was 1.5-fold more in post-OVA WT than in either KO mice. Overall, whereas proliferation of both T cell subsets to anti CD3/CD28 is comparable, response to PMA/ionomycin is somewhat compromised in KO post-OVA. (Figure 4 Panels A,B).

### Functionality of macrophages (oxidative burst response and chemotaxis to specific stimuli)

**Figure 5:**
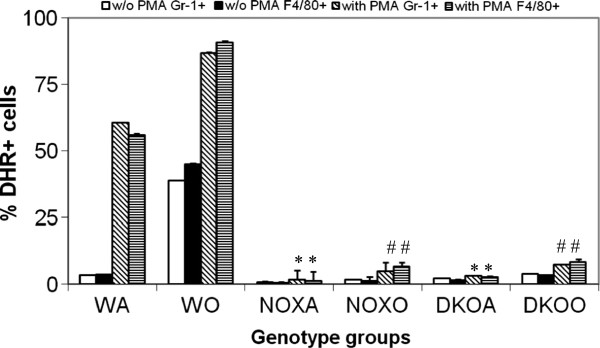
**Inhibition of oxidative burst response by KO alveolar leukocytes.** Alveolar leukocytes (0.5 × 106 cells) were stained with F4/80-Cy-Chrome and Gr1-APC for 30 min on ice, washed in PBS, warmed up at 370C for 5 min and loaded with 5mM dihydrorhodamine 123 (Molecular Probes, Eugene, OR). After 10 min at 370C, cells were split in two equal aliquots, and PMA (Sigma, St. Louis, MO) was added to one aliquot at final concentration of 1mM. After 10 min incubation cells were washed in ice-cold PBS and immediately subjected to FACS analysis. Cells were gated on neutrophils (Gr1hi), or monocyte/macrophages (F4/80+) and percentage of cells positive for dihydrorhodamine 123 fluorescence with or without PMA treatment was determined for each gate. Results shown are mean of 3 independent experiments ± SEM. (n=5/group). * denotes p value<0.05 compared to WT without PMA treatment and # denotes p value<0.05 compared to WT post-PMA treatment. While WT cells respond to PMA before as well as after OVA challenge, cells from both KO mice before as well as after OVA, failed to respond appreciably. DHR was measured at Fluorescent channel 1 in using a BD Facscaliber and DHR+ cells (CD45+gated and Gr-1+ gated or F4/80+ gated) were analyzed using CellQuestpro software.

**Figure 6:**
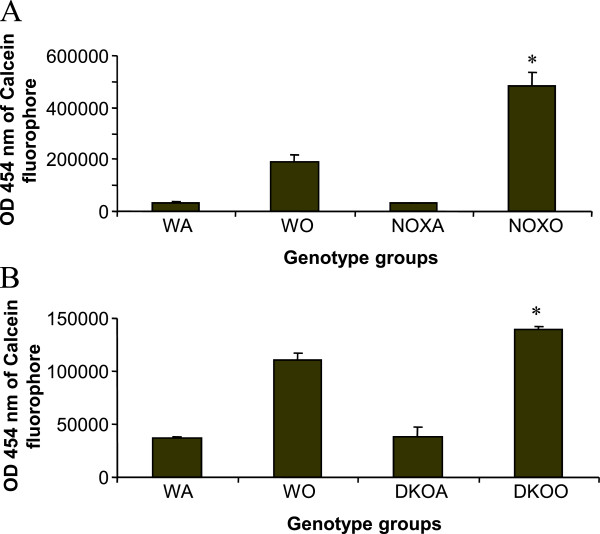
**Inhibition of MCP-1-driven chemotaxis of alveolar macrophages in post-OVA KO mice.** 15mM MCP-1 was put in 29μl volume in the lower well and 10 × 106 alveolar macrophages (from 4 mice/experimental group), also in 29μl volume in the upper wells of a 96 well Neuroprobe CTX plates (Chemicon) in high glucose medium for 2 h followed by detachment by mechanical scraping and resuspension in Phenol red-free high glucose DMEM (Gibco) with 5% FBS with 0.5μg/ml Calcein-AM (1:2000 dilution) and incubation for 20 min at 370C. Migrated cells were quantified by fluorescence (excitation at 488 nm, emission at 520 nm) using a Victor 3V (Perkin Elmer laboratories) using a Wallac1420 software. 2.5-fold and 1.26-fold increase in OD (proportinate to number of fluorescing cells in the upper well equivalent to the number of cells migrated) was found in post-OVA gp91phox−/− and DKO mice respectively. * denotes p value<0.05 compared to values in OVA-treated wildtype group.

Macrophages and neutrophils are the key downstream cells contributing to the inflammation in asthma. Their functions were measured by oxidative burst response to PMA and chemotaxis to MCP-1. Figure 5 shows drastic downregulation of DHR+ cells by FACS gated on both Gr-1+ or F4/80+ populations showing either myeloid population to be incapable of showing respiratory burst response by generating reactive oxygen species by responding to PMA. Figure 6 however, surprisingly shows upregulation calcein fluorophore (proportional to cells showing directed migration or specific chemotaxis) in both gp91phox−/− and DKO alveolar macrophages post-OVA to MCP-1.

### T cell-macrophage cross talk by mixed lymphocyte reaction (MLR)

**Figure 7:**
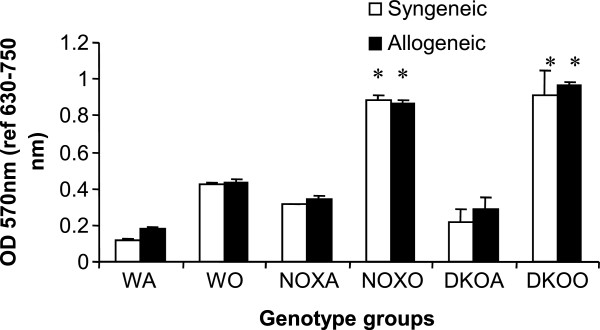
**Altered Mixed Lymphocyte Reaction in post-OVA KO mice.** 0.1 × 105 CD4+ T cells isolated by magnetic activated cell separation (MACS) by positive selection from spleen of the mice were co-cultured with γ-irradiated 0.1x103 adhering alveolar macrophages from BALf of the same experimental animal. Both cell types were from the experimental animals themselves viz. C57Bl/6 WT and KO mice. This is Syngeneic MLR where the APCs (alveolar macrophages) were γ-irradiated (3300rads). Allogeneic MLR reaction involves co-culturing 0.1x105 CD4+ T cells from the spleen of BALB/c mice with 0.1x103 γ-irradiated APCs from BALf of the experimental mice. Both control (saline-treated) and OVA-treated of each group were tested. Each culture was incubated with 1μg/ml OVA.The responders here are the T cells and the stimulators are the APCs, viz. alveolar macrophages which are γ-irradiated to inhibit their own proliferation. Since acute allergic asthma is a Th2 mediated phenomenon, interaction between T cells and macrophages will elucidate functional cross-talk between the two cell types when responders are autologous as well as when they are from a different species. 2-fold increase in post-OVA NOX vs. post-OVA WT and 2.16-fold increase in post OVA-DKO vs. post-OVA shows that T cell:APC interaction is actually more efficient in the absence of the gp91phox and MMP-12 as well as gp91phox.

Based on the aforementioned responses of T cells and macrophages it seems apparent that both cells are able to function well in response to OVA on their own at least as far as the asthma phenotype is concerned. They migrate in increased numbers from blood and resident as well as recruited cells are found in impressive inflammatory exudates around the airways. So the next question was whether there is efficient cross-talk between the T cells upstream and the macrophages downstream. To this end we did a mixed lymphocyte reaction using first the CD4+ T cells as the responders and the γ-irradiated alveolar macrophages as the stimulators from the experimental mice themselves and then used CD4+ T cells from splenocytes of BALB/c mice. Increase in proliferation measured by MTT assay (OD 570nm) (Figure 7) shows increased T cell: APC interaction both when autologous APCs (macrophages from adherent cell population in BALf of the same animal) were used and then APCs from experimental animals were used as stimulators to CD4+ T cells from spleen of BALB/c mice which were the responders.

### iNOS expression

**Figure 8:**
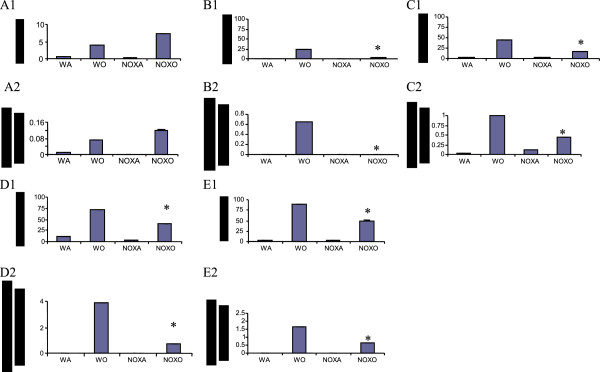
**Decreased iNOS + cells in KO mice.** Cells from all tissues viz. BM, PB, Spleen, lung and BALf of NOX−/− vs. WT with and without OVA. or we could put % and # in tabular form. **A1-E1**. Percent iNOS positive cells in BM, PB, Spleen, LP and BALf respectively. **A2-E2**. Number of iNOS positive cells in BM, PB, Spleen, LP and BALf respectively cells in million. Data shows mean of 2 independent experiments which were pooled ± SEM. (* denotes p value<0.05 compared to WO). Abbreviations used are: WT=wildtype, NOX=gp91phox−/−, WA=WT+alum, WO=WT+OVA, NOXA=gp91phox−/−+alum, NOXO=gp91phox−/−+OVA.

iNOS is a surface enzyme expressed by macrophages that are of the M1 or killer phenotype. Figure 8 shows decrease in percent iNOS+ cells in PB, spleen, lung and BALf but not BM of KO mice in comparison to corresponding tissues from WT micemay indicate that there is a skewing of macrophage phenotype from killer to healer phenotype. This corroborates well with data in Figures 5 and 6 that these macrophages although migrating to the inflammatory focus in increased numbers are incapable of typical phagocytic functions which indicates a clear dichotomy in their signaling pathways.

### Expression of co-stimulatory molecules

**Figure 9:**
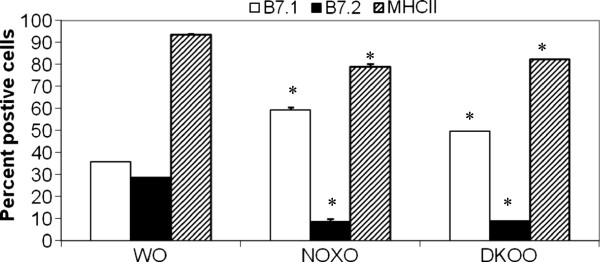
**Differential alteration of MHC and co-stimulatory molecules in KO BAL cells.** B7.1, B7.2 are co-stimulatory molecules expressed on alveolar macrophages and other antigen presentation cells like the dendritic cells and also B cells and monocytes. Expression of the said molecules were measured by FACS using specific fluorochrome conjugated antibodies from Pharmingen. The data presented shows percent cells positive for the given antigen, expressed as mean ± SEM. n=4/group.

We hypothesized that expression of MHC molecule, which controls T cell activation by APC may be somehow affected in this mechanism. Figure 9 indicates a 1.65-fold increase in post-OVA gp91phox−/− lung parenchyma cells and a 1.38-fold increase in DKO cells in B7.1 positive cells from undetected positive cells in saline treated in any group. B7.2 and MHCII expressions were however decreased in both KO mice with 3.28-fold and 3.18-fold decrease respectively in gp91phox−/− and DKO. MHCII expression was downregulated by 1.18 and 1.13-fold in the two KO mice respectively.

## Discussion and conclusions

Deletion of gp91^phox^ results in enhancement of composite asthma phenotype in mouse [17-20]. Double deletion of gp91phoix and MMP-12, a critical enzyme for phagocyte associated inflammation results in no alteration of the phenotype generated in the single deletion of gp91phox. Recruitment index (Table 1) shows statistically significant increase in recruitment of T cells in BALf of KO mice compared to WT but not in lung, while in lung parenchyma of KO mice, macrophage, PMN and basophils are preferentially upregulated compared to WT. Overall systemic response, inflammatory recruitment from blood to inflammation in lung is more in KO mice compared to WT (Table 2). Selective upregulation of both cytokine protein and cytokine mRNA of IL-13 indicates a preferential T cell mediated pathway which is unregulated in the gp91phox knockout as well as the DKO mouse. (Table 3, Figure 1) Surprisingly, there was downregulation of TGFβ which may indicate that in keeping with decreased iNOS expression and the consequent shift in macrophage phenotype to M1 (killer) from M2 (healer), TGFβ expression was also downregulated indicating a possible regulatory role for gp91phox in the development of Th2 phenotype and that deletion of the same disrupts the control or moderating effect involving cross-talk between T cells. Consequently, the phagocytes downstream that need NADPH enzyme for the respiratory burst response and proliferation are affected. Increased chemotaxis to MCP-1 may be explained by the increased expression of MCP-3. Upregulation of MMP-12 in gp91phox−/− both before and after OVA, may indicate a compensatory mechanism in the regulation of Th2 response (Table 4). Downmodulation of genes for MMP-7, 9, 10, and 28 in post-OVA SKO and DKO lungs may indicate that these metalloproteases which are also known regulators of inflammation, when downregulated in a situation of gp91phox deletion, may have disrupted a critical control mechanism on the development of Th2 mediated inflammation in lung.(Figure 2 and 3) T cells in SKO and DKO mice were functionally competent as revealed by functional tests. (Figure 4) Contradictory up- and down-modulation data of different functional responses, viz. oxidative burst response by a heterogeneous population of phagocytes in the lung and directed migration to MCP-1 gradient in a chemotaxis assay done with alveolar macrophages, indicate a dichotomy in the signaling of the same cells when different stimuli are present. (Figure 5 and 6) There was unregulated oxidative burst response (Figure 5), enhanced chemotaxis to MCP-1(Figure 6) and increased MLR (both syngeneic and allogeneic) (Figure 7), probably indicating relation to the role of these molecules in the cross-talk between T cells and alveolar macrophages, a phenomenon which can account for the “unregulated” recruitment of KO T cells to the lung interstitium which is what finally dictates development of lung inflammation in Th2 mediated allergy [16,21-25]. The overall Th2 response was enhanced possibly due to a lack of control over T cell: APC cross-talk in the KO mice as shown by the results of the MLR assay. Increased B7.1 but decreased B7.2 and MHCII expression may provide possible mechanistic insights into the regulatory function of gp91^phox^ and MMP-12 [26,27]. iNOS upregulation has always been construed as indicator of heightened inflammation by the participating cells [28-30]. Recruitment of B cells, monocytes, neutrophils and basophils are increased in lungs of both knockout mice compared to post-OVA wildtype while that of T cells, neutrophils and basophils in BALf are increased in the knockout vs. the OVA-treated wildtype (Table 2). MMP-12 controls migration of monocytes and macrophages to inflammatory sites and airway remodeling by degrading ECM proteins [31]. It is supposed to have a protective effect in emphysema [32]. B7.1, a co-stimulatory signal necessary for the activation of T cells, are expressed on cell surface by B cells, dendritic cells and macrophages, the so-called antigen presenting cells. It is associated with activation of cell mediated response, especially Th2 response. At baseline, they are not expressed but upon activation are upregulated. In our model, upregulation of B7.1 but downregulation of B7.2 and MHCII shows a possible mechanism by which gp91^phox^ and MMP-12, may synergistically regulate Th2 responsiveness and deletion of the same possibly disrupts this pathway. Mature T lymphocytes become activated to perform their effector functions when stimulated by appropriate APC bearing MHC class I or class II molecules. So antagonistic alterations in B7 family of receptors in the acute asthma pathway may indicate a definite role for either gp91phox or both gp91^phox^ and MMP-12 in controlling the co-stimulatory activating pathway in T cell activation in Th2 response.

The data presented above in this study indicate the following regulatory role for gp91^phox^ and MMP-12 in the etiology of T cell mediated acute allergic asthma pathophysiology:

(i) gp91^phox^ specifically may be regulating IL-13 gene activation in the lung tissue as well as translation into protein secreted into the airways. This may be associative.

(ii) The direction of stimulus to → response seems to be T cells to →macrophages and not vice versa. In other words, gp91^phox^ alone or gp91^phox^ and MMP-12 together regulate/translate T cell directive to macrophages for clinical manifestation of the Th2-initiated, macrophage-mediated allergic phenomenon.

(iii) Downmodulation of respiratory burst response in neutrophils and macrophages isolated from lung but upregulation of MCP-1 directed chemotaxis by alveolar macrophages collected from lung interstitium and airways, indicate a dichotomy in the role of gp91^phox^ in controlling macrophages responses to divergent stimuli in a cell specific or tissue specific manner. Upmodulated B7.1expression but downmodulateded B7.2 and MHC class II expression in KO alveolar macrophages may indicate that alteration of co-stimulatory molecule expression may give critical signals for T cell activation.

(iv) There seems to be some redundancy in their regulatory capacity for Th2 activation but gp91^*phox*^ and MMP-12 do seem to provide a regulatory checkpoint (possibly sequentially but not additively) to restrict T cell cross-talk with macrophages and keeps excessive tissue damage and ECM degradation during acute allergic inflammation under control.

## Abbreviations

WT: Wildtype; NOX: gp91^phox^ KO; OVA: Ovalbumin; AM: Alveolar macrophage; BM: Bone marrow; PB: Peripheral blood; BALf: Bronchoalveolar lavage fluid; LP: Lung parenchyma; AHR: Airway hyper-reactivity/responsiveness; i.t.: Intra-tracheal; i.v.: Intravenous; i.p.: Intraperitoneal; H&E: Hematoxylin and Eosin; Penh: Enhanced pause; WBP: Whole body plethysmography; KO: Knockout; ROS: Reactive oxygen species; NOX: NADPH oxidase; SKO: Single knockout; DKO: Double knockout; TCR: T cell receptor

## Competing interest

The authors have declared that no conflict of interest exists.

## Authors’ contributions

WRH initiated and funded the project and gave key suggestions in its execution and read this manuscript. ERB conceptualized, designed and executed all experiments and analyzed all data and wrote this manuscript. All authors read and approved the final manuscript.
